# Cathelicidin-related antimicrobial peptide protects against cardiac fibrosis in diabetic mice heart by regulating endothelial-mesenchymal transition

**DOI:** 10.7150/ijbs.35736

**Published:** 2019-09-07

**Authors:** Xiaolin Zheng, Meng Peng, Yan Li, Xule Wang, Wenjie Lu, Xi Wang, Yingguang Shan, Ran Li, Lu Gao, Chunguang Qiu

**Affiliations:** Department of Cardiology, the First Affiliated Hospital of Zhengzhou University, Zhengzhou, China

**Keywords:** Cathelicidin-related antimicrobial peptide, diabetic cardiomyopathy, EndMT, AMPKa1

## Abstract

**Cathelicidin-related antimicrobial peptide** (CRAMP), antimicrobial peptide, was reported to protect against myocardial ischemia/reperfusion injury. In the pathology of diabetic cardiomyopathy, endothelial-to-mesenchymal transition (EndMT) results from hyperglycemia-induced endothelial injury, leading to cardiac fibrosis. This study aims to evaluate the effect of CRAMP on EndMT and cardiac fibrosis on diabetic mice heart. Mice were subjected to streptozotocin to induce diabetes. CRAMP was administered by intraperitoneal injection (1 or 8 mg/kg/d) for 4 weeks from 12 weeks till 16 weeks after final streptozotocin injection. Cardiac dysfunction was observed in diabetic mice. Only 8 mg/kg/d CRAMP treatment proved cardiac function. Increased EndMT and fibrosis level were also observed in diabetic mice heart. 8mg/kg CRAMP inhibited EndMT and fibrosis level in diabetic mice. Mouse heart endothelial cells (MHECs) were treated with CRAMP and exposed to high glucose. Hyperglycemia-induced EndMT in MHECs was also attenuated by CRAMP treatment. Activation of TGFβ/Smad signalling was increased in diabetic mice heart tissue and hyperglycemia stimulated MHECs, which was prevented following CRAMP treatment. Activation of AMPKa1/mTOR showed similar changes. AMPKa1 siRNA abrogated the effects of CRAMP in MHECs. TGFβ/Smad inhibitor LY2109761 and AMPKa agonist AIRCA mimic the effect of CRAMP. In summary, CRAMP can inhibit EndMT, cardiac fibrosis and protect against diabetic cardiomyopathy by regulating AMPKa1/TGFβ signalling.

## Introduction

Cardiac fibrosis is significant feature of diabetic cardiomyopathy both in diabetes patients and animal models 1. In diabetic cardiomyopathy, extracellular matrix protein deposition and matrix cross-linking increase myocardial stiffness which mediates cardiac diastolic dysfunction 2. Myocardial fibrosis plays an important role in the pathogenesis of diabetes related heart failure. In diabetes cardiomyopathy, cardiomyocyte hypertrophy and microvascular abnormalities are often accompanied with cardiac fibrosis 3. In the pathological process of diabetes, endothelial cells (EC) are the initial targets of hyperglycemia. ECs injury plays an important role in the secretion of extracellular matrix (ECM), and promotes the development of chronic diabetic complications 4, 5. ECs undergo endothelial-to-mesenchymal transition (EndMT) after sustained damage. In this transdifferentiation process, ECs loss its' marker such as, vascular endothelial (VE)-cadherin and CD31, then obtain mesenchymal characters, such as expressing vimentin (Vim) and a-smooth muscle actin (SMA)^6^. EndMT is involved in the pathology of cardiac fibrosis in both type 1 and type2 diabetes induced cardiomyopathy 7, 8.

Transforming growth factor beta (TGFβ) is a highly effective medium widely distributed in the pathogenesis of cardiac fibrosis 9. TGF-β1 mediates cardiac fibroblast activation, ECM production, and preserves secretory phenotype in cardiac fibroblasts 10. TGFβ also regulates EndMT process by suppressing the expression of endothelial markers 11. TGFβ/Smad signal is the most important pathway to cause EndMT. Once activated, the transcription of key genes related to EndMT is actuated by smad complexes 11. Targeting on this pathway may be promising.

Cathelicidins (CRAMP in mouse/rat, LL-37 in human), designated as host defense peptides, is a major group of antimicrobial peptides (AMPs) 12. It plays essential roles in regulating host defense and immunity and serves as natural broad-spectrum antibiotics 13. Cathelicidins are produced by many cell types including immune cells, epithelial cells of the intestine, airway, skin, and urinary tract, and genital cells 12. Previous study reported that CRAMP participates in the pathogenesis of atherosclerosis in ApoE deficiency mice 14. Recently, Klyachkin YM found that CRAMP ameliorated cardiac dysfunction in myocardial infarction mice by regulating bone marrow cells retention 15. Moreover, CRAMP was reported to protect against myocardial ischemia/reperfusion injury by activation of Akt and ERK1/2^16^. These indicate the protective effects of CRAMP in cardiovascular disease. In this study, the effects of CRAMP on diabetes induced EndMT and cardiac fibrosis were evaluated.

## Methods

### Materials

Mice CRAMP purchased from Innovagen AB (Lund, Sweden). LY2109761 was from MedChem Express. Adenosine 5'-monophosphate (AMP)-activated protein kinase (AMPK) a1 siRNA were purchased from Santa Cruz. AICAR was from Sigma.

### Animal models

All of the animal care and experimental procedures conformed to the Guidelines for the Care and Use of Laboratory Animals, published by the United States National Institutes of Health (NIH Publication, revised 2011). All animal experimental procedures followed National Institutes of Health guidelines and the guidelines of our Hospital of Zhengzhou University (Approval number: ZZH 2017W08). 8-10 week-old male C57/BL6J mice were purchased from the Chinese Academy of Medical Sciences (Beijing, China). The mice diabetes model was established by streptozotocin (STZ (dissolved in 0.1 mol/l citrate buffer, pH 4.5, at a dose of 50 mg/kg for 5 consecutive days) injection as previous study described 17. The fasting blood glucose (FBG) was detected at 1 week after the final STZ injection. A FBG≥16.6 mmol/L in three independent measurements was defined as diabetes. CRAMP administered to the animals 12 weeks after the induction of diabetes by intraperitoneal injection (1 mg/kg/d or 8 mg kg/kg/d) till 16 weeks after final STZ injection. An equal volume of saline was administered to the control mice. To knock down AMPKa1, mice (C57BL6J background) were subjected to myocardial injection of lenti-shAMPKa1 at 10 weeks after the induction of diabetes.

### Adenoviral vector construction

#### Construction of recombinant lentiviruses

Recombinant lentiviruses-shAMPKa1 was constructed by Vigene Bioscience Company (Jinan, China) with AMPKa1 siRNA purchased from Santa Cruz (sc-29674). Briefly, the AMPKa1-shRNA was cloned into LentiLox 3.7. The sequence was driven by a cytomegalovirus promoter and terminated using polyadenylation signal in the 3′ long terminal repeat. The third generation packaging systems was used for lentiviral production. The lentiviral AMPKa1-shRNA and the scrambled control (shRNA) were constructed according to the standard procedure. The recombinant lentivirus was produced by transient transfection of HEK293T cells using the calcium phosphate method; the virus was harvested at 48 and 72 hours post-transfection and purified by centrifugation at 4 °C. The titer of the virus was 5× 10^10^ vp/ml.

#### Viral Delivery Protocol

Mice were receive myocardial injection of either Lenti-shAMPKa1 (1×10^10^ vp (viral particles) per animal) at 10 weeks after the final STZ injection according to our previous study described 17. Briefly, we chose left ventricular apex (1 site), anterior wall (2 sites), and lateral wall (2 sites). Each site was injected with 10μl lentivirus vector (1×10^10^ vp) by using a 29-gauge syringe.

### Echocardiography and hemodynamics

After anaesthetized by 1.5% isoflurane, mice were subjected to echocardiography measurement by using MyLab 30CV ultrasound (Biosound Esaote), as described previously 18, 19. The left ventricular (LV) end-diastolic diameter (LVEDd), LV end-systolic diameter (LVESd), LV ejection fraction (LVEF), and LV ejection of shortening (LVFS) were analysed.

After anaesthetized by 1.5% isoflurane, mice were subjected to hemodynamics measurement by using cardiac catheterization, as described previously 18, 19. PVAN data analysis software was used to process data. The heart rate (HR), maximal rate of pressure development (dp/dtmax), maximal rate of pressure decay (dp/dtmin), and time constant of LV pressure decay (Tau) were analysed.

### Histological analysis

H&E, Picrosirius Red staining, immunohistochemical analysis and immunofluorescence staining were performed according to our previous studies 18, 20. The LV collagen volume was visualized using light microscopy (Olympus FSX100; Olympus Corporation, Tokyo, Japan). The following antibodies were used for immunohistochemical and immunofluorescence staining: Wheat germ agglutinin (WGA), collagen III, CD31, and α-SMA.

### Cell culture and treatment

Primary adult mouse heart ECs (MHECs) was isolated as previous study described 8. Briefly, 4-6 week mouse hearts were cut in Hanks' balanced salt solution buffer. Collagenase A was used to digest heart tissue. 10% FBS-DMEM was used to stop digestion. After filtered by a nylon mesh (70-mm pores), cells were re-suspended in the Hanks' solution. CD31 beads were used to bind ECs. After being washed, ECs were cultured in dishes pre-coated with 2% gelatin (Sigma, Oakville, ON, Canada) in endothelial basal medium with 10% FBS with a density of 1×10^5^ cells/ml. After cultured in serum-free media for 24 h, MHECs were cultured with 33 mM glucose (high glucose, HG) for 48 h in the presence or absence of different concentrations of CRAMP (10, 25, 50, 100 μg/L). Cells in the control group were exposed to normal glucose concentration (NG; 5.5 mM glucose) and 27.5 mM mannitol to control for osmolarity. Each experiment was performed three times independently. MTT assay was used to detect cell viability. To inhibit TGFβ signaling, LY2109761 (0.1μM) was used. To inhibit AMPKa1, AMPKa1 siRNA were used. To agonist AMPKa, AICAR (1 mM) was used.

Neonatal rat cardiomyocytes (NRCMs) were isolated according to previous study. Briefly, 1- to 3-day-old Sprague-Dawley rat hearts were harvested. Ventricles were digested four times for fifteen min each in 0.125% trypsin-EDTA in PBS. Following centrifugation, cells were resuspended and incubated for 90 min in a 100-mm dish to allow noncardiac myocytes (mainly cardiac fibroblasts) to adhere to the plastic. NRCMs and cardiac fibroblasts were treated with HG and CRAMP (100 μg/L) for 24 h.

### Scratch adhesion test

Mouse heart endothelial cells (MHECs) were plated in 6-well plates. After treated with HG and/or CRAMP (10, 30 μM) for 48 h, MHECs were cultured in serum-free media for 4 h. A 200 μl sterile micropipette tip was used to scrap cells to create wounds. Migratory cells were photographed at 0, 12, 24, and 48 h after scraping. Mobility = (area at T0 - area at T48) / area at T0 * 100%.

### Immunofluorescence

The immunofluorescence staining was performed as our previous study described 21. MHECs were incubated with primary antibodies against VE-cadherin (Abcam, ab33168) and vimentin (Santa Cruz, sc-5565). Cardiac fibroblasts were incubated with primary antibodies against α-SMA (Abcam).

### RT-PCR and Western blot

RT-PCR and Western blot were performed according to our previous studies 17, 19. Total RNA was extracted from frozen, pulverized mouse cardiac tissue using TRIzol™ (Roche Diagnostics, Mannheim, Germany). The RNA (2 µg of each sample) was reverse-transcribed into cDNA using oligo(dT) primers and the Transcriptor First Strand cDNA Synthesis kit (Roche Diagnostics). The PCR products were quantified using a LightCycler 480 SYBR® Green 1 Master Mix (04707516001; Roche Diagnostics). Following an initial 5 min denaturation step at 95˚C, a total of 42 primer-extension cycles were carried out. Each cycle consisted of a 10 sec denaturation step at 95˚C, a 20 sec annealing step at 60˚C, and a 20 sec incubation at 72˚C for extension. Then a final extension step was performed at 72 ˚C for 10 min. The double standard curve was used to quantify the PCR results. The results were normalized against GAPDH gene expression. The sequences of the oligonucleotide primers (Sangon Biotech, Shanghai, China) were as in Table 1.

Heart tissue or cells were lysed in radioimmunoprecipitation (RIPA) lysis buffer and 50 µg cell lysate was used for protein separation by 10% SDS-PAGE. The proteins were then transferred to polyvinylidene difluoride (PVDF) membranes (Millipore). Specific protein expression levels were normalized to the GAPDH protein levels of the total cell lysate and cytosolic proteins on the same PVDF membranes. The following primary antibodies were used: CD31, VE-cadherin, collagen I, collagen III, α-SMA, TGFβ and total AMPKa1 (purchased form Abcam, 1:1000 diluted), smad4, phosphorylated (P-) and total mammalian target of tapamycin (mTOR, Ser2448), and P-AMPKa1 (Ser485) (purchased form Cell Signaling Technology, 1:1000 diluted). Antibody incubation was performed overnight with gentle shaking at 4˚C. Quantifcation of the western blots was performed using an Odyssey infrared imaging system (LI-COR Biosciences, Lincoln, NE, USA). The secondary antibodies, goat anti-rabbit IRdye® 800 CW (LI-COR) IgG and goat anti-mouse IRdye® 800 CW (LI-COR), were used. The blots were scanned using an infrared Li-Cor scanner. The results were normalized against GAPDH gene expression.

### Elisa

Serum and hearts sample from mouse 0, 2, 4 weeks after injection were taken for measurement of the level of CRAMP peptide using the mouse CRAMP ELISA kit (CUSABIO, CSB-E15061m).

### Statistical analysis

SPSS 23.0 was used for data analysis. The results are expressed as the means ± SD. Two-way analysis of variance (ANOVA) followed by the post hoc LSD test was used to analyse comparisons in groups. Student's unpaired t-test was used to detect comparisons between two groups. All experiments were performed blinded. P value less than 0.05 was defined as statistical significance.

## Result

### CRAMP improves cardiac function in diabetic mice

**Figure 1:**
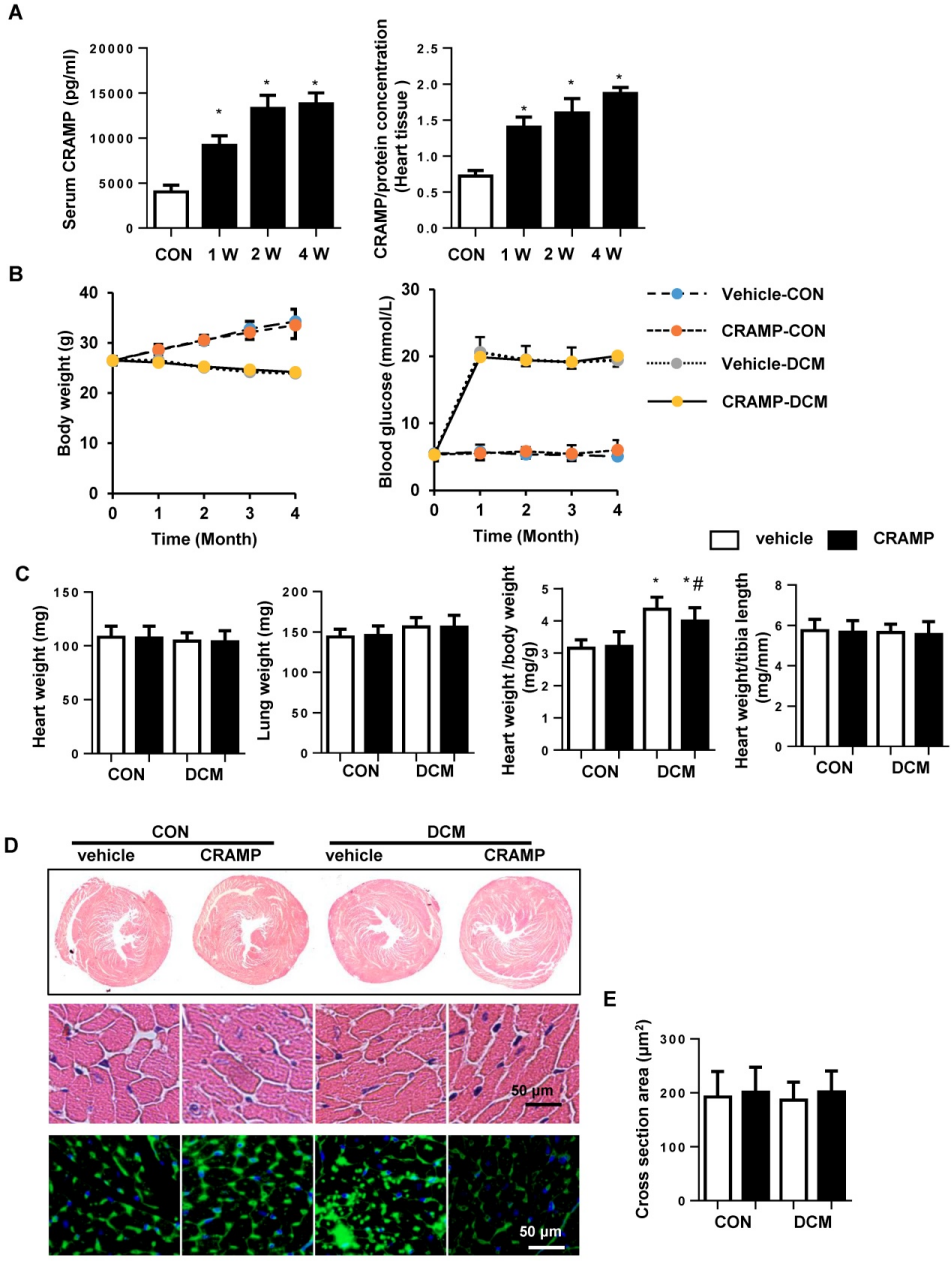
** CRAMP improves cardiac function in diabetic mice.** A. CRAMP level in serum and myocardium after 1, 2, 4 weeks of CRAMP injection (8mg/kg/d)(n=6, *P<0.05 vs. CON). B. Body weight and blood glucose of mice at 0, 1, 2, 3, 4 months after STZ injection (n=10). C. Heart weight, lung weight, heart weight to body weight ratio, and heart weight to tibia length ratio in diabetic mice treated with CRAMP (n=10). D. Representative image of the heart with H&E staining and wheat germ agglutinin (WGA) staining (n=6). E. The cell surface area of cardiomyocytes in heart tissue (n=100+ cells per group). *P<0.05 vs the corresponding Sham; #P<0.05 vs. vehicle-DCM.

To investigate the effect of CRAMP on cardiac dysfunction during diabetes pathology, mice were treated with 1 or 8mg/kg CRAMP. The Echocardiography and hemodynamics results revealed that 4 months after STZ injection, systolic and diastolic dysfunction were observed in vehicle-treated diabetic mice, as evidenced by increased Tau value, and reduced LVEF, LVFS, decreased dp/dt_max_ and dp/dt_min_, and increased Tau value. Both 1mg/kg and 8mg/kg CRAMP treatment did not affect heart rate in both physical condition and diabetic mice. But only 8mg/kg CRAMP improved cardiac function as assessed by increased LVEF, LVFS, augmented dp/dt_max_ and dp/dt_min_ and reduced Tau value compared with that in vehicle-DCM group (Table 2). Thus we chose 8mg/kg CRAMP to the further study. The CRAMP was increased in serum and myocardium after 1, 2, 4 weeks of injection (Fig. 1A).The body weight was increased in sham mice and decreased in diabetic mice. The blood glucose was increased diabetic mice compared with sham group. The body weight and blood glucose were no significant difference between CRAMP-treated mice and vehicle-treated mice in both physical condition and diabetic status (Fig. 1B). The heart weight, lung weight, heart weight to tibia length ratio and the cross section area (CSA) were no significant difference among four groups (Fig.1 C-E), but the heart weight to body weight ratio was increased in vehicle-DCM mice and reduced sharply in CRAMP-treated group (Fig. 1C).

### CRAMP inhibits cardiac fibrosis in diabetic mice

**Figure 2:**
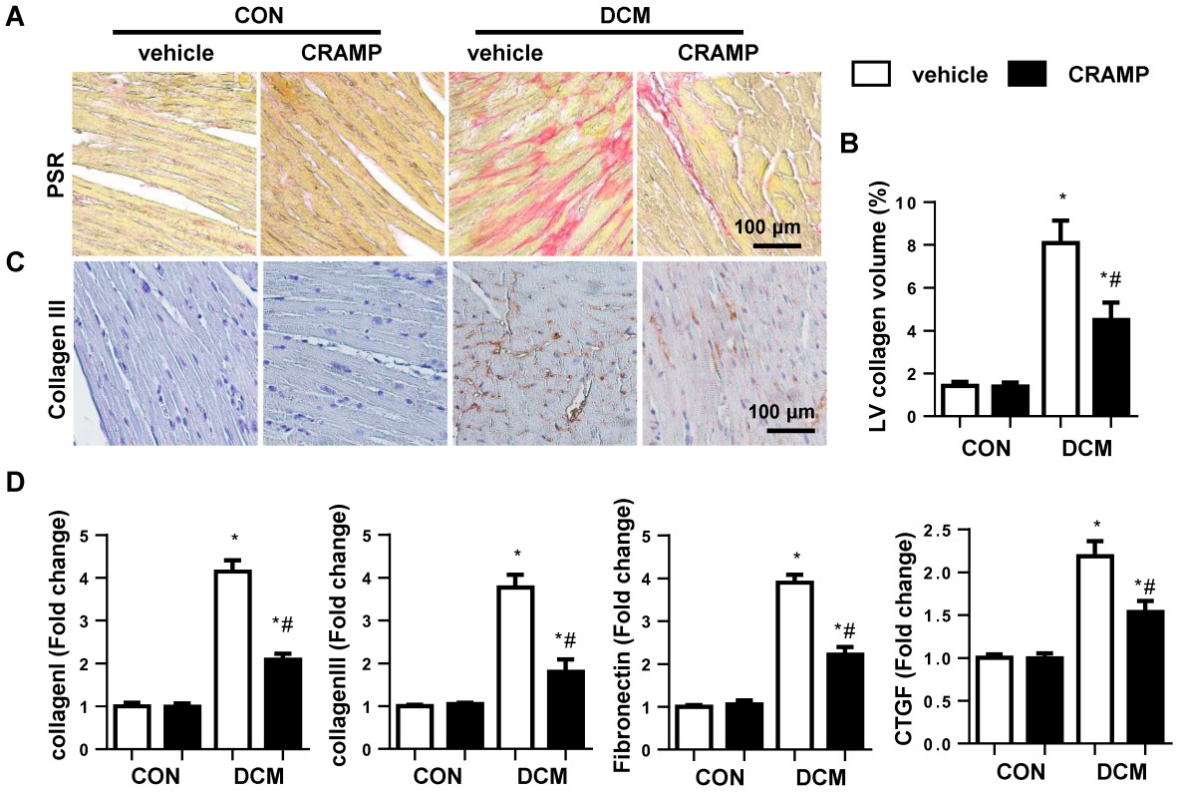
** CRAMP inhibits cardiac fibrosis in diabetic mice.** A and C. Representative image of the heart with Picro-Sirius red (PSR) staining and Immunohistochemical staining of collagen III (n=6). B. Quantification of the total collagen volume in the indicated group. D. PCR analysis of fibrotic markers (collagen I, collagen III, TGFβ, CTGF) in diabetic heart tissue (n=6). *P<0.05 vs the corresponding Sham; #P<0.05 vs vehicle-DCM.

The cardiac fibrosis was assessed by using PSR staining and transcription level of fibrotic markers. Increased LV collagen volume (8.07%±1.06) and transcription level of fibrotic markers including collagen I, collagen III, connection tissue growth factor (CTGF), and fibronectin were observed in diabetic mice heart (Fig. 2A-D). While LV collagen volume (4.50%±0.79) and the transcription level of fibrotic markers were reduced in CRAMP-treated diabetic mice (Fig. 2A-D).

### CRAMP decreases EndMT in diabetic mice

**Figure 3:**
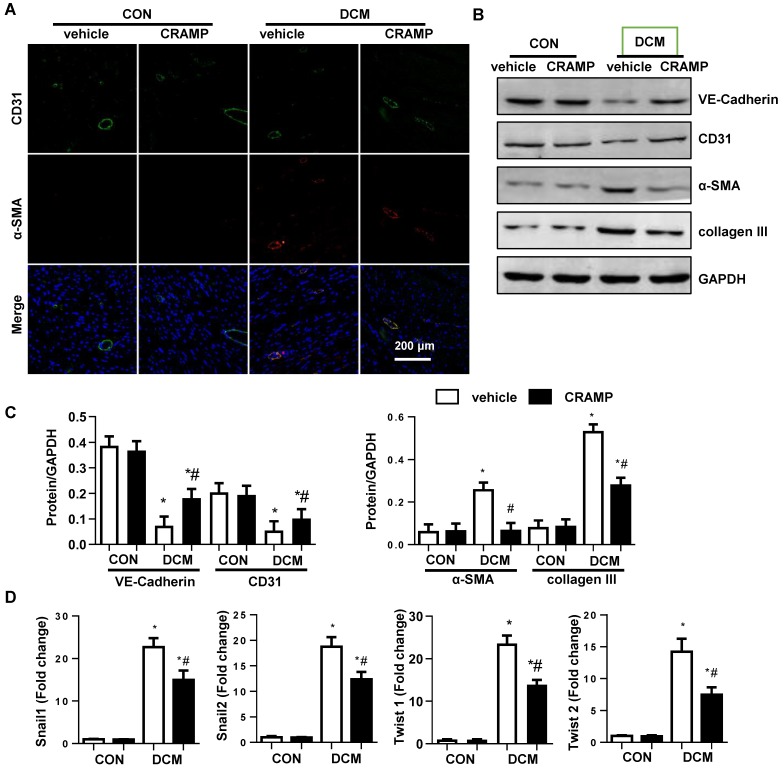
** CRAMP decreases EndMT in diabetic mice.** A. Immunofluorescence staining of CD31 and α-SMA in diabetic hearts (n=6). B and C. Representative western blot (B) and analysis (C) of CD31, VE-cadherin, α-SMA, collagen III in diabetic hearts (n=6). G. PCR analysis of EndMT markers (snial1, snial2, twist1, twist2) in diabetic heart tissue (n=6). *P<0.05 vs the corresponding Sham; #P<0.05 vs vehicle-DCM.

An increase expression level in fibroblast markers (collagen I, collagen III, vimentin) and decreased expression level in endothelial markers (CD31 and cadherin) were observed in vehicle-treated mice heart compared with sham mice (Fig. 3A-C). The transcription level of EndMT markers (snial1, snial2, twist1, and twist2) was also increased in diabetic mice (Fig. 3D). CRAMP treatment reduced these EndMT transition phenotypes (Fig. 3A-D).

### CRAMP attenuates high glucose induced EndMT in MHECs

**Figure 4:**
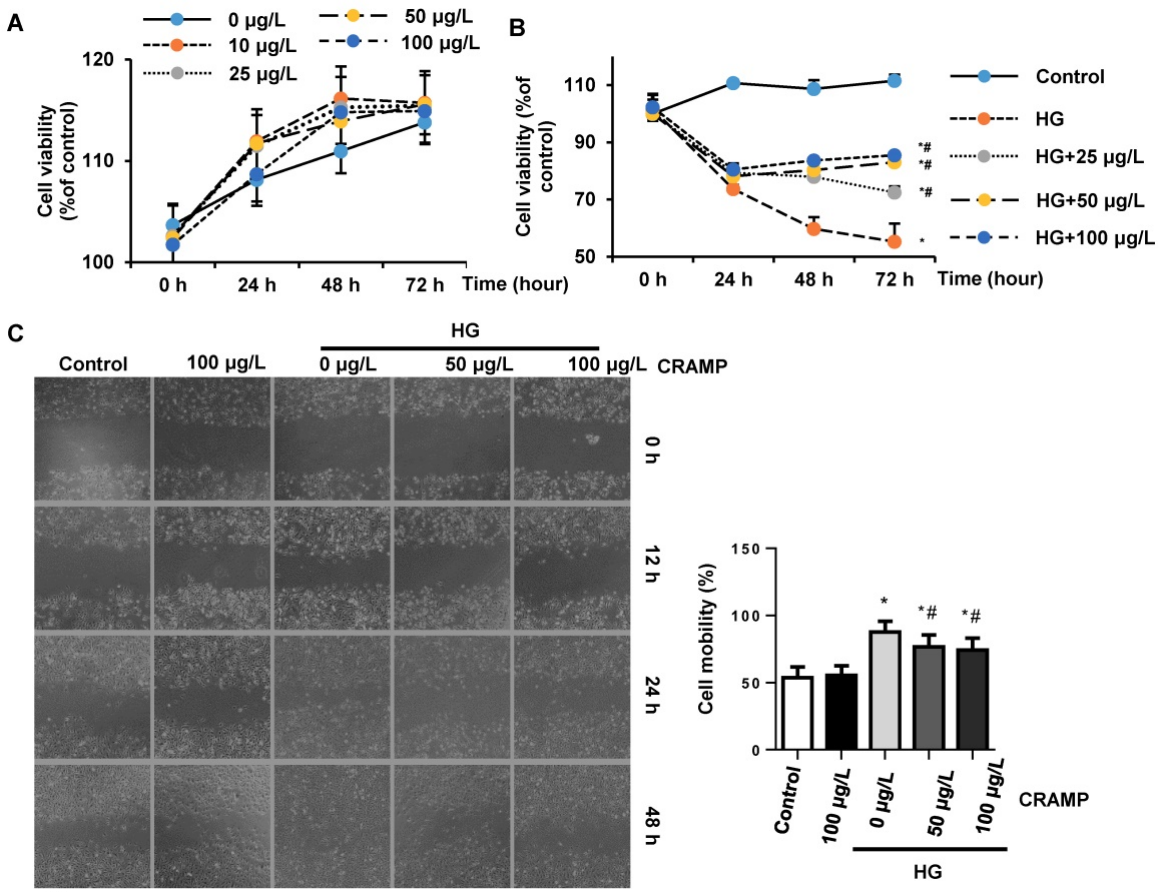

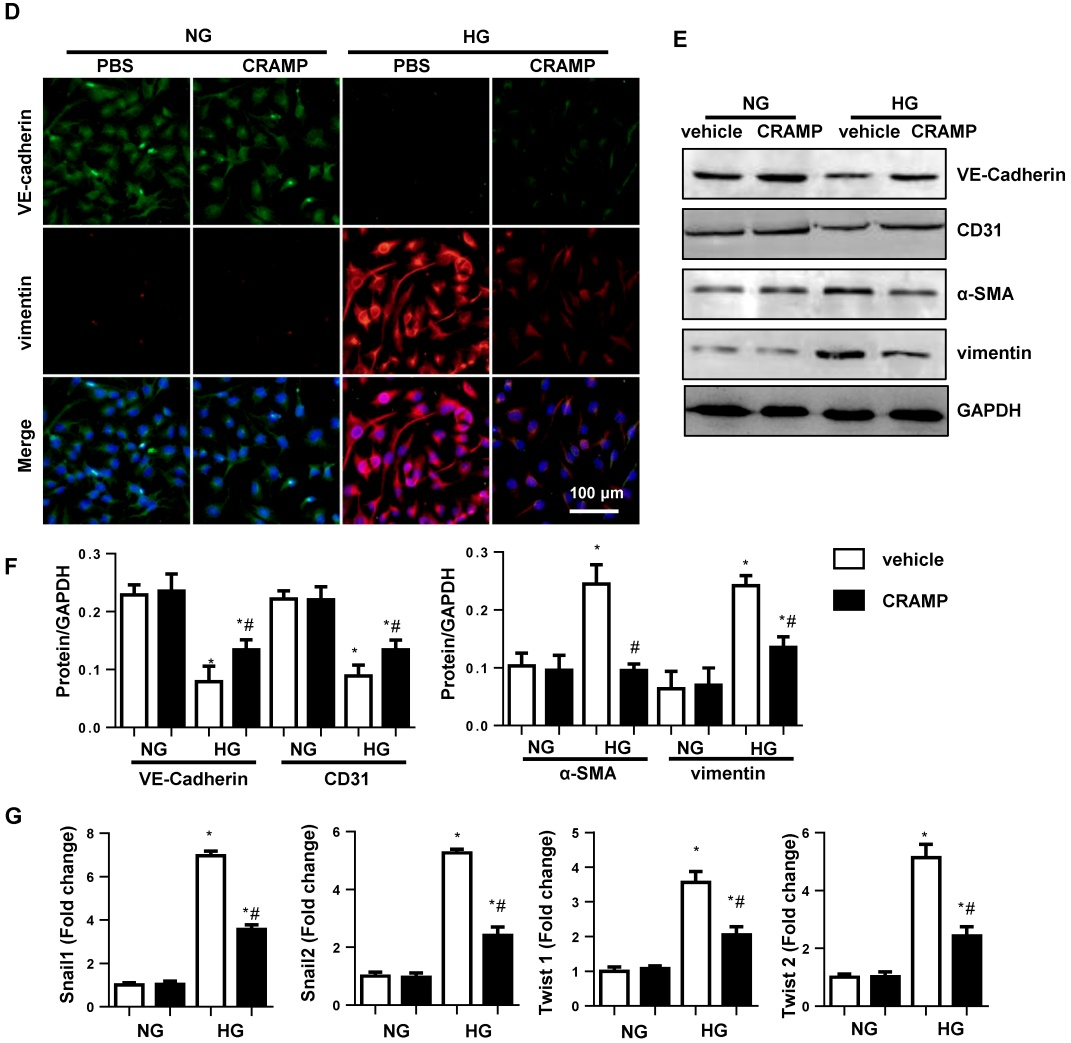
** CRAMP attenuates high glucose induced EndMT in MHECs.** A. Cell viability in MHECs treated with different concentrations of CRAMP (0, 10, 25, 50, 100 μg/L) (n=6 sample). B. Cell viability in MHECs treated with different concentrations of CRAMP (0, 10, 25, 50, 100 μg/L) under HG condition (n=6 samples). C. Cell migration ability detected by scratch assay after cells were treated with CRAMP (50 or 100 μg/L) and exposed to HG (n=6 samples). D. Immunofluorescence staining of VE-cadherin and vimentin in MHECs (n=6 samples). E and F. Representative western blot (E) and analysis (F) of CD31, VE-cadherin, α-SMA, and vimentin after cells were treated with CRAMP (100 μg/L) and exposed to HG (n=6 samples). G. PCR analysis of EndMT markers (snial1, snial2, twist1, twist2) in MHECs (n=6 samples). *P<0.05 vs the corresponding NG; #P<0.05 vs vehicle-HG.

To confirm the direct effect of CRAMP on endothelial cells, MHECs were isolated and cultured with high glucose in the presence of CRAMP (10, 25, 50, 100 μg/L). MTT result revealed that different concentrations of CRAMP (10, 25, 50, 100 μg/L) did not affect cell viability (Fig. 4A). HG stimulation induced decreased cell viability, while CRAMP increased cell viability in a dose-and time- dependent manner (Fig. 4B). To test the cell migration, cells were scraped. The cell migration rate was increased in HG stimulated group but decreased in CRAMP treated group (Fig 4C). HG induced increased EndMT in MHECs as assessed by increased expression level of fibrotic markers (α-SMA, vimentin) and decreased expression level of endothelial markers (CD31, VE-cadehrin) (Fig. 4D-F). 50 and 100 μg/L CRAMP reduced these transition (Fig 4D-F). The increased transcription level of EndMT markers (snail1, snail2, twist1, and twist2) was reduced by CRAMP treatment (Fig 4G).

### CRAMP inhibits TGFβ/Smad signaling in diabetic heart and high glucose stimulated MHECs

**Figure 5:**
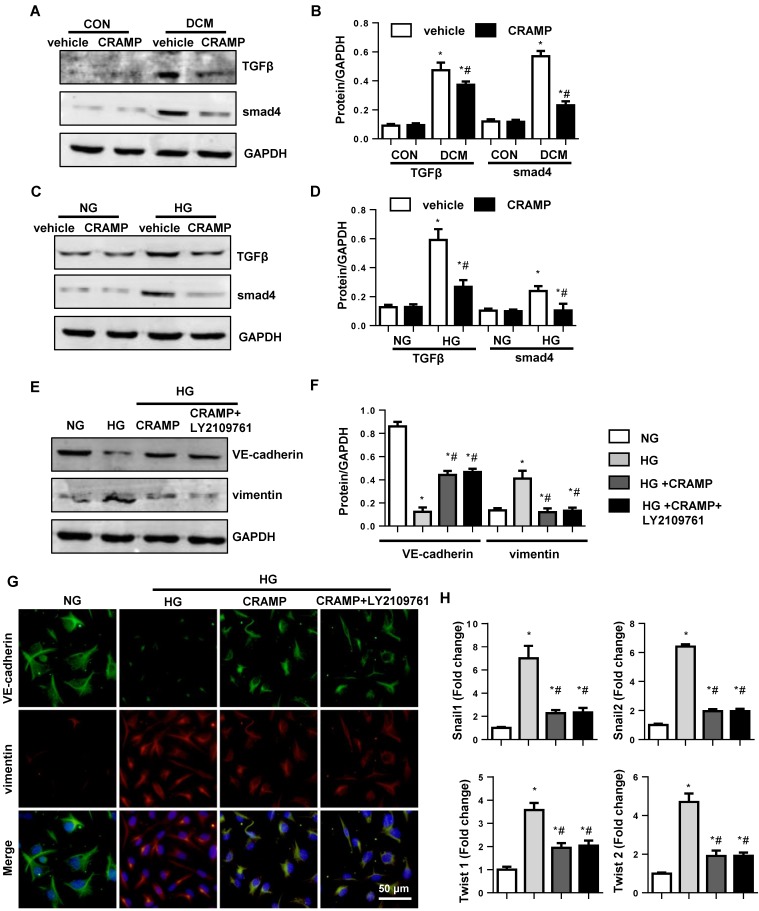
** CRAMP inhibits TGFβ/Smad signaling in diabetic heart and high glucose stimulated MHECs**. A and B. Representative western blot (A) and analysis (B) of TGFβ, smad4, in diabetic mice treated with CRAMP (n=6). *P<0.05 vs the corresponding Sham; #P<0.05 vs vehicle-DCM. C and D. Representative western blot (C) and analysis (D) of TGFβ, smad4 in MHECs treated with CRAMP (30 μM) (n=6 samples). *P<0.05 vs the corresponding NG; #P<0.05 vs vehicle-HG. E-H. MHECs were treated with CRAMP (100 μg/L) and LY2109761 (0.1μM), and exposed to HG. E and F. the expression level of VE-cadherin and vimentin in the indicated group (n=6 samples). G. Immunofluorescence staining of VE-cadherin and vimentin in the indicated group (n=6 samples). H. PCR analysis of EndMT markers (snial1, snial2, twist1, twist2) in MHECs (n=6 samples). *P<0.05 vs the control; #P<0.05 vs HG.

Since TGFβ/Smad signal is the most important pathway to cause EndMT, we detected the TGFβ signaling. As expected, the expression of TGFβ and activation of smad4 were increased in both diabetic mice heart and HG stimulated MHECs. CRAMP (100 μg/L) decreased the expression level of TGFβ and activation of smad4 in both diabetic mice heart (Fig. 5A, B) and HG stimulated MHECs (Fig. 5C, D). To confirm whether TGFβ/Smad signaling is the only target of CRAMP, cells were incubated with TGFβ/Smad inhibitor, LY2109761. As a result, CRAMP suppressed EndMT as assessed by increased expression level of VE-cadherin, increased expression of vimentin, and reduced transcription of snail1, snail2, twist1, and twsit2, while LY2109761 did not further improve these effects (Fig. 5E-H). These indicate CRAMP exerts its effects via targeting on of TGFβ signaling.

### The effects of CRAMP on AMPKa signaling

**Figure 6:**
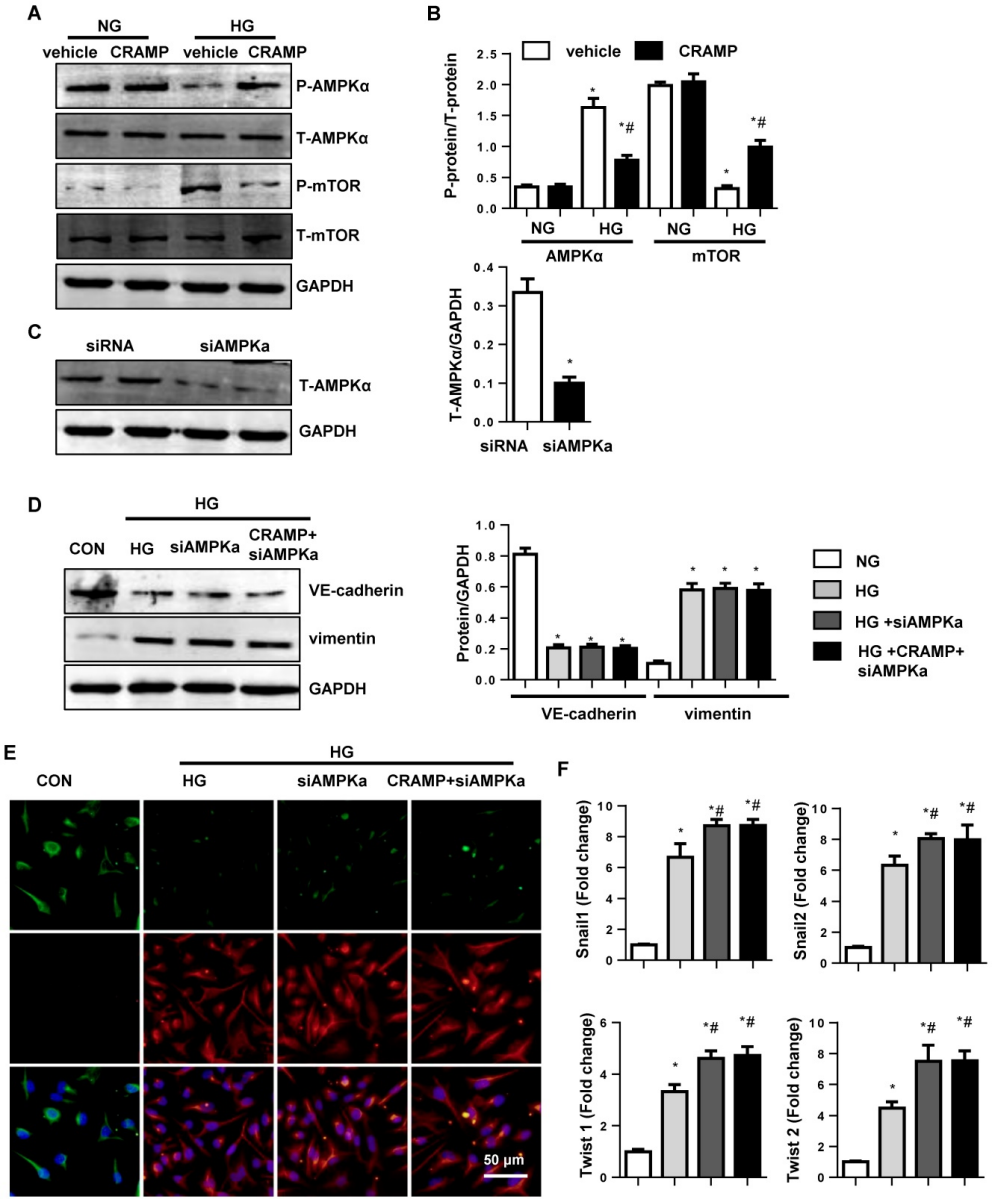
** The effects of CRAMP on AMPKa signalling.** A and B. Representative western blot (A) and analysis (B) of P-AMPKa1, T-AMPKa1 P-mTOR, and T-mTOR in MHECs treated with CRAMP (100 μg/L) (n=6 samples). *P<0.05 vs the corresponding NG; #P<0.05 vs vehicle-HG. C-F. MHECs were treated with CRAMP (100 μg/L) and AMPKa1 siRNA and exposed to HG. C. The expression of AMPKa1 after cells were treated with siRNA (n=6 samples). D. The expression level of VE-cadherin and vimentin in the indicated group (n=6 samples). E. Immunofluorescence staining of VE-cadherin and vimentin in the indicated group (n=6 samples). F. PCR analysis of EndMT markers (snial1, snial2, twist1, twist2) in MHECs (n=6 samples). *P<0.05 vs the siRNA; #P<0.05 vs HG-siRNA.

Our previous study reported that CRAMP inhibited fibroblast activation by activating AMPKa signalling. Thus, we detected the AMPKa signalling on MHECs. Consistently, we found that the phosphorylation level of AMPKa was reduced in both diabetic mice heart and HG stimulated MHECs. Consistently, the phosphorylation level of down-stream protein mTOR was increased in both diabetic mice heart and HG stimulated MHECs compared with vehicle-treated MHECs. While, CRAMP increased the phosphorylation level of AMPKa, reduced the phosphorylation level of mTOR in HG stimulated MHECs (Fig. 6A, B). To confirm whether TGFβ/Smad is regulated by AMPKa, cells were treated with AMPKa1 siRNA (Fig. 6C). The EndMT level in cells treated with AMPKa1 siRNA was no significant difference with that in HG group as evidenced by the same expression level of VE-cadherin, vimentin, and the transcription level of snail1, snail2, twist1, and twist2 when compared with that in HG group. While CRAMP could not remove the increased EndMT after cells treated with AMPKa1 siRNA (Fig. 6D-F). These indicate AMPKa knock down abrogates the protective effects of CRAMP.

### CRAMP enhance the anti-EndMT effects of AMPKa agonist AICAR

**Figure 7:**
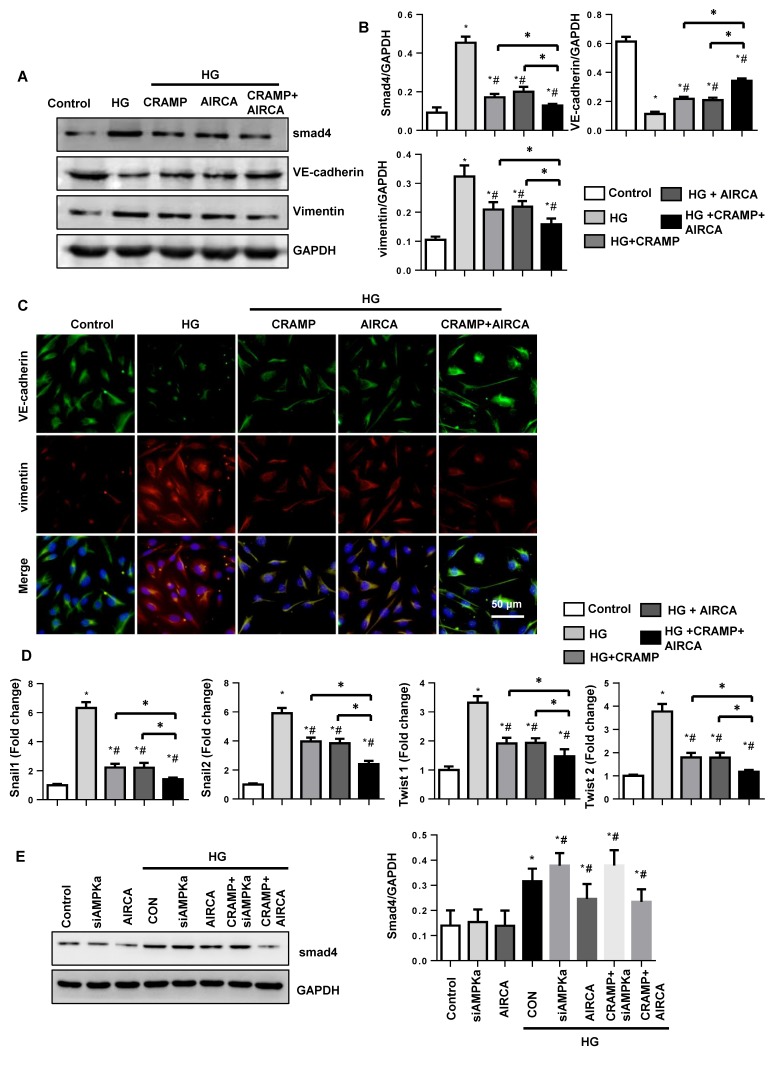
** CRAMP enhances the anti-EndMT effects of AMPKa agonist AICAR.** MHECs were treated with CRAMP (100 μg/L) and AMPKa agonist (AICAR) and exposed to HG. A and B. The expression level of smad4, VE-cadherin and vimentin in the indicated group (n=6 samples). C. Immunofluorescence staining of VE-cadherin and vimentin in the indicated group (n=6 samples). D. PCR analysis of EndMT markers (snial1, snial2, twist1, twist2) in MHECs (n=6 samples). E. Expression of smad4 in the indicated group (n=6). *P<0.05 vs the control; #P<0.05 vs HG.

Then the next question is whether CRAMP could augment the anti-EmdMT effect of AMPKa agonist. To solve this question, we then used AMPKa agonist, AICAR. AICAR decreased smad4 activation and exert anti-EndMT effect as CRAMP as evidenced by the increased expression of VE-cadherin, and decreased expression of vimentin, and reduced transcription level of snail1, snail2, twist1, and twist2 when compared with that in HG group. CRAMP further improved these effects as evidenced by the increased expression of VE-cadherin, and decreased expression of vimentin, and reduced transcription level of snail1, snail2, twist1, and twist2 when compared with that in AICAR-HG group (Fig. 7A-D). Altogether, these data suggest CRAMP exert anti-EndMT effects via regulating AMPKa/TGFβ signaling and could enhance the effects of AMPKa agonist AICAR.

To evaluate whether TGFβ inhibition was reliant on AMPKa activation, MHECs were treated with AMPKa1 siRNA and AIRCA. As shown in Figure 7E, smad4 was elevated in HG group. Under HG stimuli, AMPKa1 siRNA increased the expression of smad4, while AIRCA reduced the expression of smad4. And CRAMP could not reduce the elevated smad4 in AMPKa1 knockdowm cells. These indicated that TGFβ/Smad inhibition was reliant on AMPKa activation.

### AMPKa1 knock down abrogates the protective effects of CRAMP

**Figure 8:**
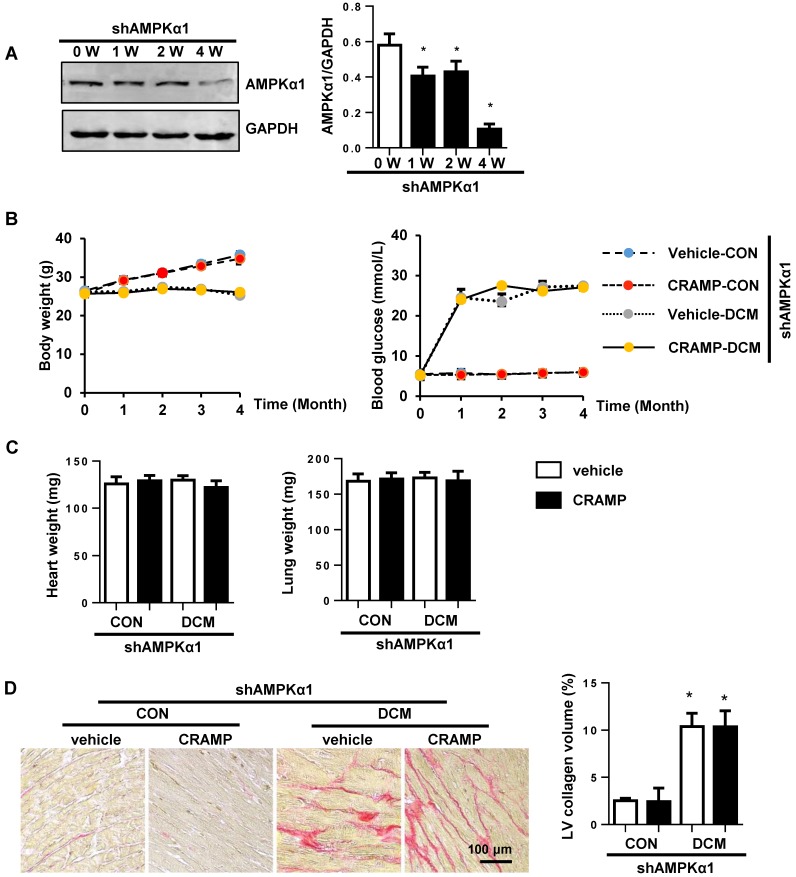

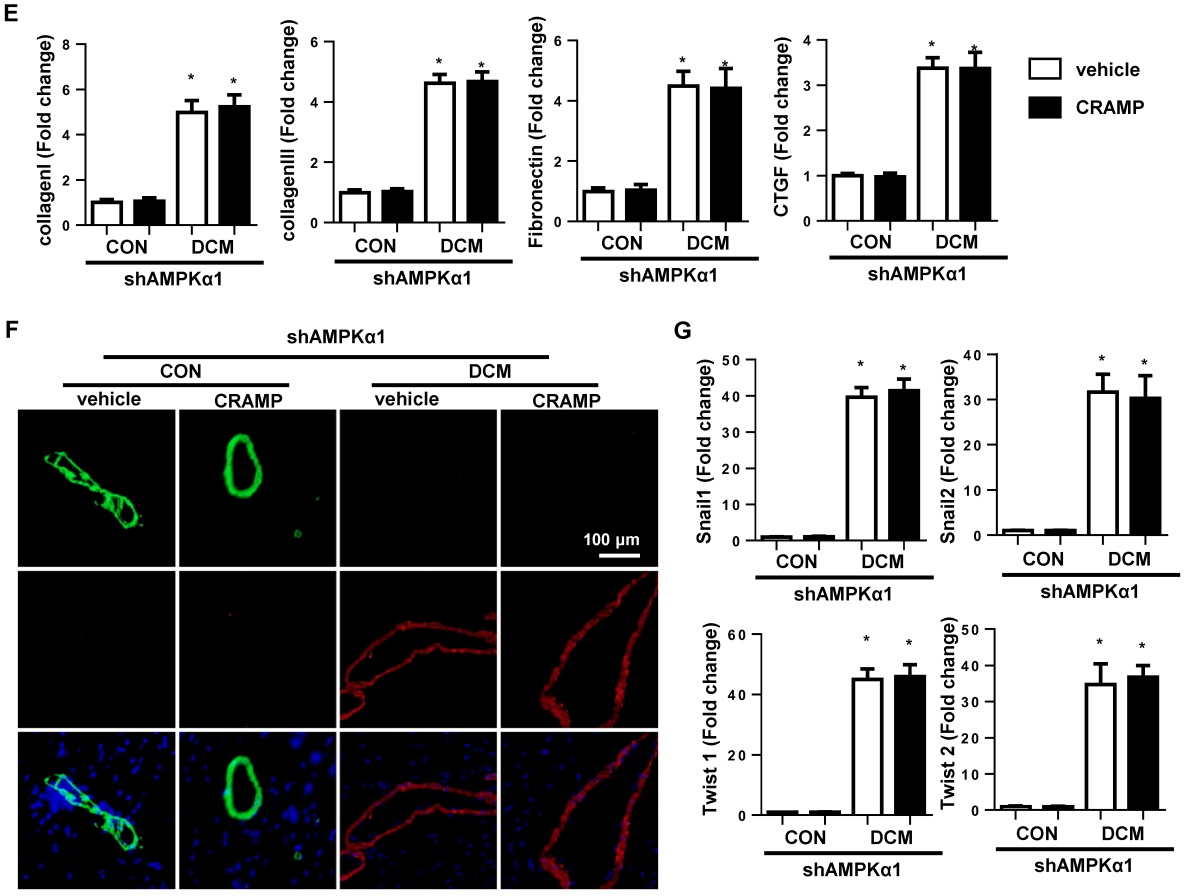
** AMPKa1 knock down abrogates the protective effects of CRAMP.** Diabetic mice were subjected to myocardial injection of lenti-shAMPKa1 and treated with CRAMP. A. The expression level of AMPKa1 in heart 0, 1, 2, 4 weeks after injection of lenti-shAMPKa1 (n=6). B. Body weight of mice at 0, 1, 2, 3, 4 months after STZ injection (n=10). D. Blood glucose and body weight of mice at 0, 1, 2, 3, 4 months after STZ injection (n=10). D. Representative image of the heart with Picro-Sirius red (PSR) staining (n=6) and Quantification of the total collagen volume in the indicated group. E. PCR analysis of fibrotic markers (collagen I, collagen III, TGFβ, CTGF) in diabetic heart tissue (n=6). F. Immunofluorescence staining of CD31 and α-SMA in diabetic hearts (n=6). G. PCR analysis of EndMT markers (snial1, snial2, twist1, twist2) in diabetic heart tissue (n=6). *P<0.05 vs the corresponding sham; #P<0.05 *vs.* vehicle-DCM.

Then the next question is whether CRAMP protects diabetic cardiomyopathy via AMPKa induced anti-EndMT effects. To solve this question, mice were subjected to myocardial injection of lenti-shAMPKa1 to knockdown AMPKa1 (Fig. 8A). There was no significant difference in body weight and blood glucose between vehicle-treated diabetic mice and CRAMP-treated diabetic mice under AMPKa1 knock down condition (Fig. 8B). There was also no significant difference in heart weight and lung weight among the four groups (Fig. 8C). AMPKa1 knock down abolished the anti-fibrosis (Fig. 8D, E) and anti-EndMT effects (Fig. 8F, G) of CRAMP. There was no significant difference in cardiac dysfunction between CRAMP-treated diabetic mice and vehicle-treated diabetic mice after AMPKa1 knock down (Table 3). These data confirm that the anti-fibrosis effect of CRAMP was relying on its anti-EndMT effects, which was mediated by regulating of AMPKα1-TGFβ1 signaling in endothelial cells.

### CRAMP on cardiomyocytes and cardaic fibroblasts

**Figure 9:**
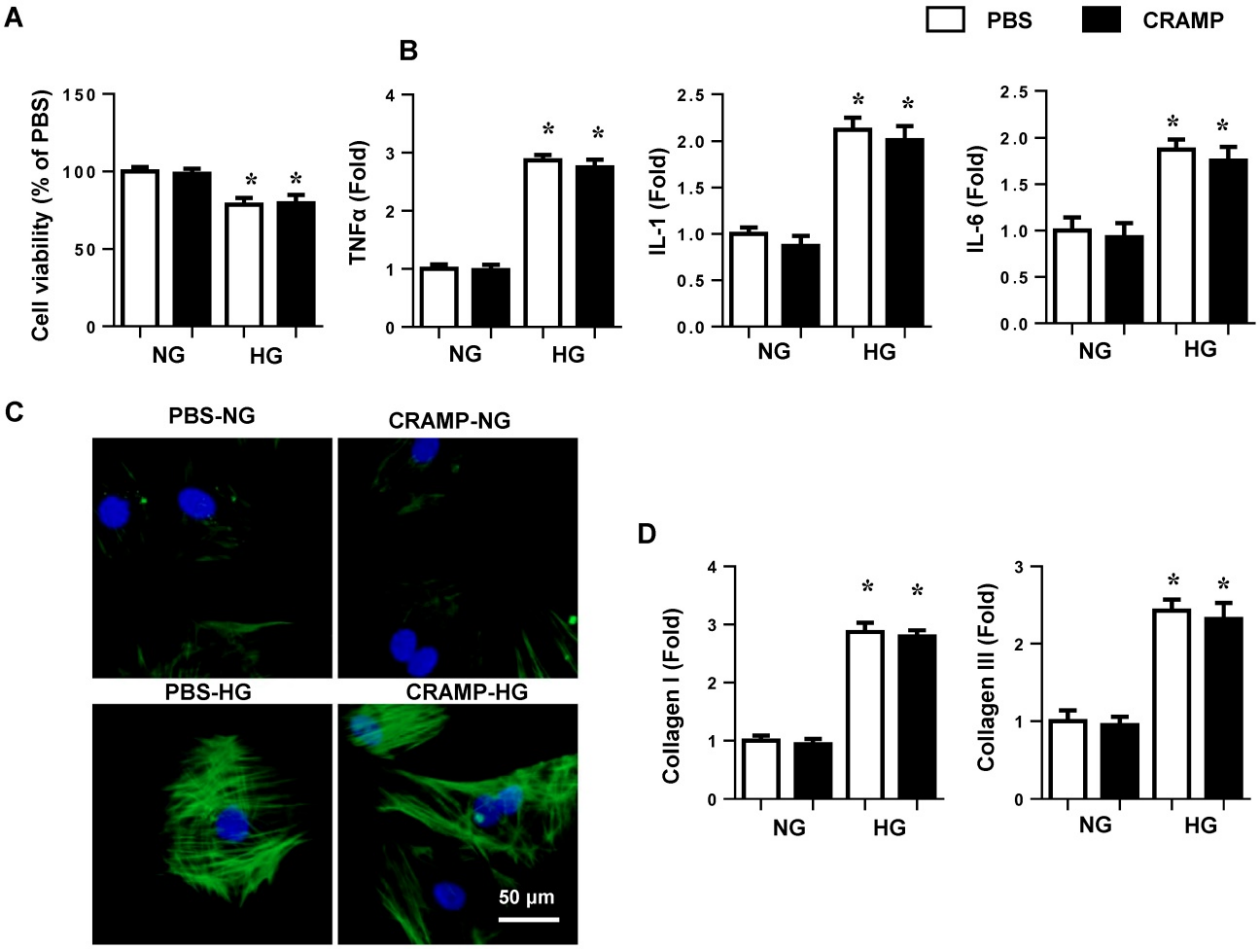
** CRAMP on cardiomyocytes and cardiac fibroblasts.** A and B. NRCMs were treated with HG and CRAMP (100 μg/L) for 24h. A. cell viability (n=5). B. mRNA expression of TNFα, IL-1 and IL-6 (n=6). C and D. Cardiac fibroblasts were treated with HG and CRAMP (100 μg/L) for 24h. C. α-SMA staining (n=5). D. mRNA expression of collagen I and collagen III (n=6).

To elucidate the effects of CRAMP on cardiomyocytes and cardiac fibroblasts, NRCMs and neonatal rat fibroblasts were isolated and stimulated with HG for 24h. HG induced reduced cell viability and increased cell inflammation in NRCMs. CRAMP did not affect cell viability and cell inflammation in NRCMs (Fig. 9A, B). Cardiac fibroblasts were stimulated with HG for 24h. HG induced increased cell α-SMA expression and increased mRNA expression of collagen I and collagen III, while CRAMP did not affected these α-SMA and collagen expression in cardiac fibroblasts (Fig. 9 C, D).

## Discussion

Evidence implicating EndMT in cardiac fibrosis has been mounting for several years. In a landmark publication in 2007, Kalluri et al. demonstrated that EndMT makes a significant contribution to myocardial fibrosis in the adult heart (27%-33%) 6. Then EndMT/EMT has proved to be associated with cardiac fibrosis in many other cardiovascular disease models such as hypertrophic cardiomyopathy and diabetes-induced heart disease 7. During the pathology of diabetes, ECs are one of the earliest cell types that are exposed to hyperglycemia 8. Hyperglycemia interlinked metabolic abnormalities, which lead to ECs damage and change. These damage lead to a complex network of gene activation and repression programs. In this process, ECs change their polarity, morphology, functionality and cell-cell interaction to adopt a mesenchymal phenotype 11. Studies have proved the benefit of inhibiting EndMT in diabetic cardiomyopathy 7, 8. In the current study, we show that diabetes-induced EndMT is increased with increased fibrosis level. EndMT is also observed in high glucose stimulated MHECs. CRAMP inhibited these phenotypic changes in endothelial cells and prevents diabetes-induced cardiac functional abnormalities.

TGFβ plays a crucial role in cardaic fibrosis by regulating cardiac fibroblast activation and ECs EndMT process 9. Smad3 deficiency or the systemic inhibition of TGFβ, decreased EndMT in ECs and ameliorated cardiac fibrosis in vivo 22, 23. In keeping with our first finding, we show that CRAMP modulates EndMT through inhibition of TGFβ/Smad pathway. TGFβ/Smad inhibitor could mimic the anti-EndMT effects of CRAMP. Combination treatment with CRAMP and TGFβ/Smad inhibitor did not further improved the anti-EndMT effects, suggesting TGFβ/Smad is the only pathway that affected by CRAMP.

AMPK is a serine threonine kinase, which acts as a fuel gauge in the process of cell stress to maintain energy balance. 24. AMPK functions in diabetes, cancer and cardiovascular disease 24. Under physiological condition, vascular AMPK guarantees energy supply to modulate vascular function and blood flow 25. However, when ECs expose to sustained hyperglycemia, vascular AMPK undergoes chronic deactivation, which shifts the balance to metabolic disorder, leading to ECs damage 8. Recently, studies have reported that AMPK activation could inhibit TGFβ pathway. Zheng W reported that AMPK agonist Metformin prevents peritendinous fibrosis by inhibiting TGFβ signaling 26. Xiao Y found that by activation of AMPK, TGF-β/smad pathway was inhibited, which mediated the protective effect of baicalin on pressure overload induced cardiac fibrosis 27. Moreover, Hinson JT et al. have found a crosstalk between AMPK and post-transcriptional regulation of TGFβ signaling that has implications in fibrotic forms of cardiomyopathy 28. In our study, we found that CRAMP's effect on TGFβ inhibition was reliant on AMPKa activation in MHECs. Considering that endothelial cells express both α1 and α2 subunits, and AMPKα1 is predominates in ECs, we silence AMPKα1 subunits by AMPKα1 siRNA. AMPKa1 knock down increased smad4 activation, which totally abolished CRAMP's anti-EndMT effects both in vivo and vitro. While AMPKa agonist AIRCA inhibited smad4 activation and mimic CRAMP's anti-EndMT effects in MHECs. Our in vitro study confirmed that CRAMP could further enhance the anti-EndMT of AIRCA, indicating that combination use of CRAMP and AIRCA may provide a new therapeutic method to treat DCM to reduce the side effect of AIRCA.

Studies have reported that mice were observed an increased myocytes size after 2-5 months of STZ injection 29, 30. But in our study, we did not observe this change in myocytes size in DCM group (4 months after STZ injection). A reduced body weight was observed in two DCM groups, which may attribute to the unchanged myocytes size since the heart weight keeps proportion with the body weight. This inconsistence need to be further elucided. The anti-fibrosis effect may acount for the protevtive effects on cardiomyocytes and fibroblasts. In our study, we did not find any protective effects of CRAMP on HG stimulated cardiomyocyted inflammation and fibroblast activation. Thus, the protective effect of CRAMP on DCM heart mainly relies on endothelial cells.

**Figure 10:**
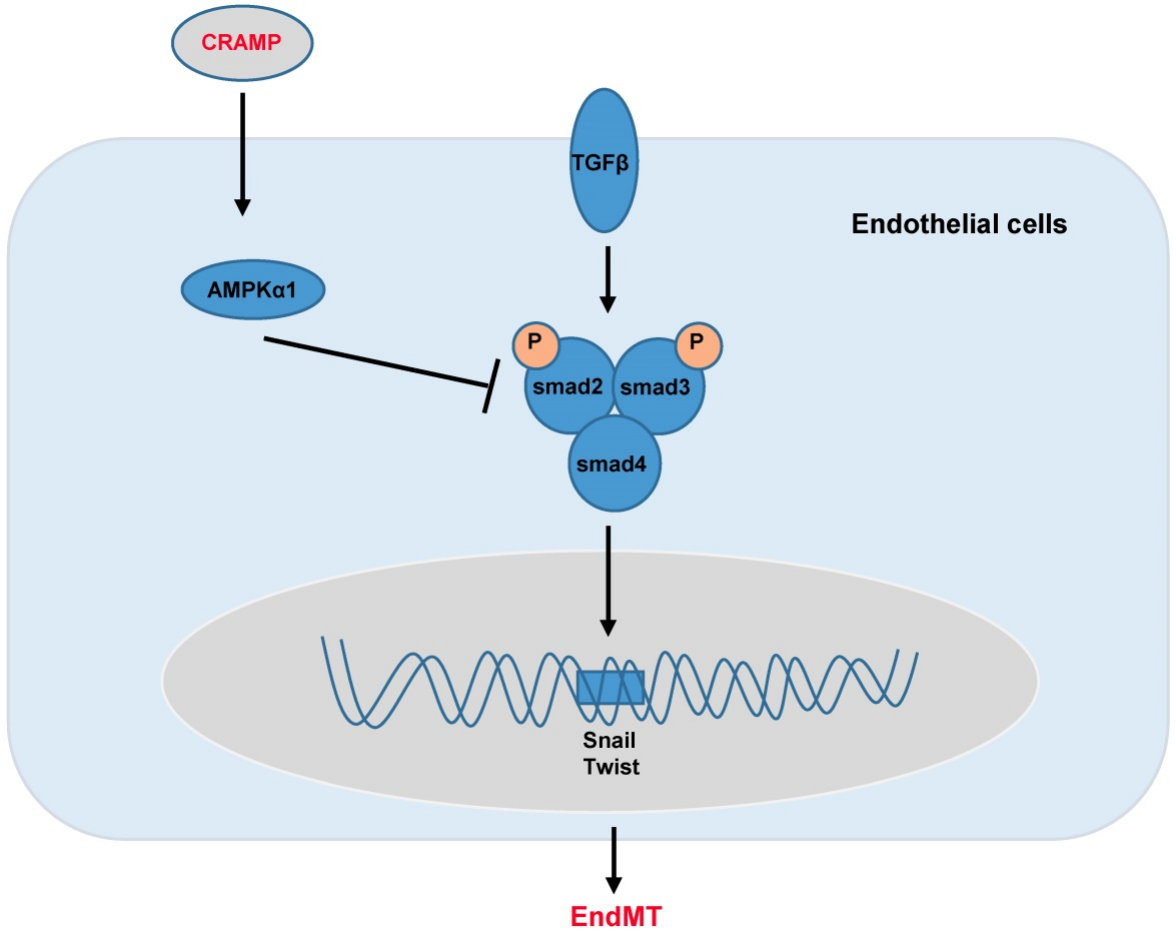
** Graphic abstract.** CRAMP protects cardiac fibrosis in diabetic mice heart

In summary, we found that CRAMP protected diabetic cardiomyopathy via AMPKα1-dependent regulation of the TGFβ pathway (Fig. 10). Our study implicates that CRAMP could become a promising medicine for the treatment of diabetic cardiomyopathy.

## Figures and Tables

**Table 1:** All primers used in our study

Primers	Forward	Reverse
Collagen I^m^	AGGCTTCAGTGGTTTGGATG	CACCAACAGCACCATCGTTA
Collagen III^m^	AAGGCTGCAAGATGGATGCT	GTGCTTACGTGGGACAGTCA
Fibronectin^m^	CCGGTGGCTGTCAGTCAGA	CCGTTCCCACTGCTGATTTATC
CTGF^m^	AGGGCCTCTTCTGCGATTTC	CTTTGGAAGGACTCACCGCT
Snail1^m^	CCAAACCCACTCGGATGTGA	TCTTGGTGCTTGTGGAGCAA
Snail2^m^	TTCTACGTTCTCTGGGCTGG	GCAGTGAGGGCAAGAGAAAG
Twist1^m^	TCGGACAAGCTGAGCAAGAT	CCAGACGGAGAAGGCGTAG
Twist2^m^	GCTACAGCAAGAAATCGAGCG	CTGCAGCTCCTCGAAAGACT
GAPDH^m^	ACTCCACTCACGGCAAATTC	TCTCCATGGTGGTGAAGACA
Collagen I^r^	GAGAGAGCATGACCGATGGATT	TGGACATTAGGCGCAGGAA
Collagen III^r^	AAGGGCAGGGAACAACTGAT	GTGAAGCAGGGTGAGAAGAAAC
TNFa^r^	AGCATGATCCGAGATGTGGAA	TAGACAGAAGAGCGTGGTGGC
IL-1^r^	GGGATGATGACGACCTGCTAG	ACCACTTGTTGGCTTATGTTCTG
IL-6^r^	GTTGCCTTCTTGGGACTGATG	ATACTGGTCTGTTGTGGGTGGT
GAPDH^ r^	GACATGCCGCCTGGAGAAAC	AGCCCAGGATGCCCTTTAGT

Sequences are listed 5'-3'. m Primers used in mouse experiments. r Primers used for rat cardiomyocytes and neonatal rat cardiac fibroblasts.

**Table 2:** Echocardiography and hemodynamics Parameters in diabetic mice after treated with CRAMP.

	Vehicle-CON (n=8-10)	CRAMP-CON (n=8-10)	Vehicle-DCM (n=10)	CRAMP (LD)-DCM (n=10)	CRAMP (HD)-DCM (n=10)
LVEF (%)	68.0±6.1	66. 4±5.6	42.0±8.9*	43.7±10.1*	53.1±7.3*#
LVFS (%)	31.8±4.3	30.7±3.8	16.8±4.2*	17.7±4.9*	22.5±3.8*#
HR (bpm)	452±21	446±27	441±58	478±60	453±39
dp/dt max (mmHg/s)	9491±761	9496±789	4501±577*	4712±515*	6905±750*#
dp/dt min(mmHg/s)	-9056±849	-9065±693	-4330±682*	-4807±892*	-6818±968*#
Tau (Weiss; ms)	8.34±1.29	9.24±1.06	18.83±1.59*	16.6±1.52*	13.94±1.44*#

**Table 3:** Echocardiography and hemodynamics Parameters in diabetic mice after injected with Ad-shAMPKα1 and treated with CRAMP.

	Vehicle-CON (n=8)	CRAMP-CON (n=8)	Vehicle-DCM (n=8)	CRAMP -DCM (n=8)
LVEF (%)	61.8±3.9	62.1±3.6	38.6±3.1*	38.5±4.7*
LVFS (%)	30.6±1.7	29.8±3.9	14.9±2.5*	15.4±1.9*#
HR (bpm)	485±46	496±50	496±69	486±53
dp/dt max (mmHg/s)	8394±481	8497±561	3745±438*	3776±439*
dp/dt min(mmHg/s)	-8975±766	-8736±819	-3851±807*	-3945±629*
Tau (Weiss; ms)	9.28±1.34	8.87±1.37	22.23±3.19*	23.13±2.02*

LVEF, left ventricular ejection fraction; LVFS, left ventricular ejection of shortening; HR, heart rate; dp/dtmax, maximal rate of pressure development; dp/dtmin, maximal rate of pressure decay; Tau, time constant of LV pressure decay *P<0.05 for difference from corresponding sham group. #P<0.05 vs vehicle-DCM group.
